# Feasibility and preliminary efficacy of a systematic transaction model-guided dyadic coping nursing intervention for patients with breast cancer and their spousal caregivers: A pilot study

**DOI:** 10.1016/j.apjon.2024.100621

**Published:** 2024-11-14

**Authors:** Yuan Wang, Jiajia Zhang, Shan Wang, Yibo Wu, Ling Hang, Yuming Hua, Weifeng Shi

**Affiliations:** a Human Reproductive and Genetic Center, Affiliated Hospital of Jiangnan University, Wuxi, China; b Wuxi Furen Senior High School, Wuxi, China; c Department of Breast Surgery, Affiliated Hospital of Jiangnan University, Wuxi, China

**Keywords:** Breast cancer, Dyadic coping, Body image, Post-traumatic growth, Pilot study, Spousal caregiver

## Abstract

**Objective:**

This study aimed to assess the feasibility, acceptability, and preliminary efficacy of a Systematic Transaction Model (STM)-guided dyadic coping nursing intervention for patients with breast cancer and their spouses.

**Methods:**

A single-arm, pre-test/post-test pilot study was conducted at a tertiary hospital in Wuxi, China, recruiting 28 breast cancer patient–caregiver pairs. Each dyad participated in six hybrid intervention sessions. Paired t-tests were used to evaluate pre- and post-intervention changes, and effect sizes were calculated. Feasibility was assessed by recruitment and retention rates, acceptability via the Client Satisfaction Questionnaire-8 (CSQ-8), and preliminary efficacy through measures of body image, dyadic coping, post-traumatic growth, and marital satisfaction.

**Results:**

All 28 dyads completed the intervention. Patients showed small-to-moderate improvements in body image, post-traumatic growth, dyadic coping, and marital satisfaction (*d* ​= ​0.4–0.5, *P* ​≤ ​0.022), with clinically meaningful changes observed in 39%–68% of patients. Spousal caregivers also demonstrated improvements in post-traumatic growth, dyadic coping, and marital satisfaction (*d* ​= ​0.3–0.6, *P* ​≤ ​0.033), with 36%–46% showing clinically important differences.

**Conclusions:**

This pilot study supports the feasibility and initial efficacy of an STM-guided dyadic coping intervention, which may benefit breast cancer patients and their spouses as a unit. Further large-scale trials are recommended to validate these findings.

**Trial registration:**

China Clinical Trial Registry (ChiCTR2400083416).

## Introduction

According to the latest data from the International Agency for Research on Cancer (IARC), female breast cancer is the second leading cause of cancer incidence worldwide in 2022, with an estimated 2.3 million new cases, or 11.6% of all cancer cases. Of these, 666,000 will die, accounting for 6.9% of all cancer deaths.^1^ The diagnosis of breast cancer often results in the onset of numerous physical and psychosocial issues.^2^ Surgical treatment results in the loss of one or both breasts, which can lead to a perceived loss of femininity and glamour. This can cause patients to be reluctant to look at their bodies directly. Additionally, surgical procedures may result in the formation of scars, swelling, redness of the skin, and lymphoedema, including body image disturbances, traumatic stress syndrome, fatigue, and psychological depression and anxiety.^3^ These complications have a profound impact on the quality of life of individuals diagnosed with breast cancer.^4^ In the context of married individuals diagnosed with breast cancer, the spouse assuming the role of primary caregiver is confronted with a multitude of challenges, including the burden of extensive caregiving responsibilities, financial constraints, and internalized role conflicts.^5^ The experience of breast cancer has a profound impact on couples, leading to diminished resilience to the disease in both partners.^6^ This is evidenced by lower levels of body image, marital satisfaction, dyadic coping, and post-traumatic growth.

According to previous studies, the prevalence of body intention disorder among women treated for breast cancer was 52.5%.^7^ Patients with body image disorder are disinclined to divulge their thoughts and feelings to their spouses due to the emergence of negative mentalities such as low self-esteem and the stigma associated with the condition. This can result in a sense of disconnection and suspicion between the two parties, which in turn impedes the expression of dyadic behaviors within the couple.^8^ Body intention disorder not only increases the psychological burden of patients, but also harms their partners, leading to a decrease in intimacy and affecting the marital relationship between couples.^9^ In addition, a diagnosis of breast cancer is a major traumatic event, which is associated with a more complex set of stressors than traditional trauma.^10^ The diagnosis of cancer and the financial stress of treatment can cause negative psychological distress for both the patient and the spouse,^11^ however, as the disease improves after treatment, the couple makes positive psychological changes that are referred to as post-traumatic growth (PTG),^12^ i.e., the process of positive psychological change experienced after struggling with a traumatic event.^13^ Post-traumatic growth has a buffering effect on distress and is associated with positive psychological health in cancer patients and their spouses' positive mental health.^14^ Previous studies have shown that increasing post-traumatic growth in breast cancer patients and spouses is beneficial for maintaining a good couple relationship and promoting marital satisfaction for both spouses.^15^ In our preceding study, we discovered that the levels of body image, dyadic coping, and post-traumatic growth in breast cancer patients were interrelated. Furthermore, we found that body image indirectly affects post-traumatic growth through dyadic coping. In other words, when a certain level of dyadic coping exists, body image can have an impact on post-traumatic growth.^16^ Accordingly, we devised a couple-based dyadic intervention program with the objective of enhancing the patient's level of body image, which in turn facilitates post-traumatic growth and ultimately enhances marital satisfaction in the couple.

In the face of the stress of cancer, couples often use a variety of coping strategies, such as individual coping and dyadic coping (DC), to mitigate the negative effects of the stressor.^17^^,^^18^ In contrast to individual coping, dyadic coping demonstrates the duality with which couples deal with the stressor, i.e. couples deal with the stressor as an interacting unit, rather than isolated individuals. The available evidence from studies of cancer couples indicates a significant relationship between DC and couples' ability to adapt to the challenges of a cancer diagnosis. A systematic review has highlighted the significance of DC in maintaining and, in some cases, enhancing marital functioning in couples who are dealing with a cancer diagnosis.^19^ Previous studies have shown that dyadic interventions based on Chinese colorectal cancer couples have achieved favorable results in improving marital satisfaction, post-traumatic growth, and quality of life.^20^^,^^21^ The existence of such relationships between DC and cancer adaptation provides a rationale for interventions aimed at improving DC, which may also benefit cancer couples' adaptation to cancer.^22^ In previous studies, supportive interventions aimed at improving couples' dyadic coping have been developed in the field of oncology.^23^, ^24^ However, these interventions have been conducted primarily in Europe and the United States.^19^ Researchers need to develop more dyadic coping interventions for couples facing other types of cancer (e.g., breast cancer) in other countries (e.g., Asian countries). Therefore, our research team developed a couple-based dyadic coping intervention program for patients with breast cancer and spousal caregivers to validate its feasibility and preliminary efficacy.

In light of the necessity for a dyadic theoretical framework to more effectively direct the advancement of a couple-based intervention for couples managing cancer, the Systemic Transactional Model (STM) for DC in couple dyads were selected as the theoretical foundation of our intervention program.^25^ Bodenman's STM posits that both partners' stressful experiences, coping efforts, and stress management outcomes are intertwined, and thus both partners' well-being and satisfaction are dependent on a shared process of stressful coping regulation.^26^ The STM hypothesizes that a partner's experience of stress (which affects the partner's mood, well-being, and behavior) always affects, directly or indirectly, both partners in a committed relationship ("we stress").^27^ In addition, STM suggests that positive dyadic coping can maintain or re-establish internal stability between the patient and his or her spouse, strengthening communication, cohesion, and relationship satisfaction for both spouses, thus promoting positive physiological and psychological changes for the patient.^28^ STM posits that shared processes of stress and coping regulation exert considerable influence over the form of DC, which, in turn, is associated with the outcomes of the couple's experience, including marital satisfaction, post-traumatic growth and body image.^25^

Therefore, we selected dyadic coping based on the Systemic Transactional Model of Guidance as the theoretical basis for the intervention program. An intervention program to improve the level of body image (patient), dyadic coping, post-traumatic growth, and marital satisfaction (couple) was developed for patients with breast cancer and spousal caregivers to validate the feasibility, acceptability, and preliminary effects of the program in breast cancer couples.

## Methods

This was a single-arm before-and-after pilot trial. A total of 28 breast cancer patient-partner dyads completed a baseline survey and received 6 sessions intervention; participants completed a follow-up survey one week after the last intervention. Feasibility was assessed through retention and session completion rates. The Client Satisfaction Questionnaire (CSQ-8) was used to assess acceptability of the program during the follow-up survey.^29^ Initial efficacy was assessed through effect sizes of psychosocial health outcomes assessed at baseline and follow-up surveys. The research staff consisted of a psychologist, two clinical nurses, and five graduate students. The purpose, significance, and content of this study were explained to each patient-partner dyad prior to the start of the study. Uniformly trained investigators used a uniform questionnaire in the survey and surveyed participants for baseline information through a face-to-face method.

### Participants

The study was conducted between January 2024 and May 2024. Convenience sampling method was used to recruit subjects. All subjects were recruited from the Department of Breast Surgery, Affiliated Hospital of Jiangnan University, Wuxi, Jiangsu Province, China, and the inclusion criteria were as follows: (1) both spouses were ≥ 18 years old; (2) the female partner had a pathologic diagnosis of breast cancer; (3) the female partner had received surgical treatment; (4) both spouses were married, and their spouses were the primary caretakers and lived together; and (5) they were clearly conscious, with good reading and comprehension skills. Exclusion criteria: (1) both spouses had received psychotherapy; (2) one of them had other malignant tumors or severe cardiac, hepatic, and renal dysfunction. Study subjects were selected based on rigorous inclusion and exclusion criteria.

### Ethical statement

The study design was approved by the Ethics Committee of the Affiliated Hospital of Jiangnan University in Wuxi, Jiangsu Province, China (IRB No. LS2023073). Each participant signed an informed consent form, and patient privacy was protected throughout the study.

### Interventions

**Fig. 1:**
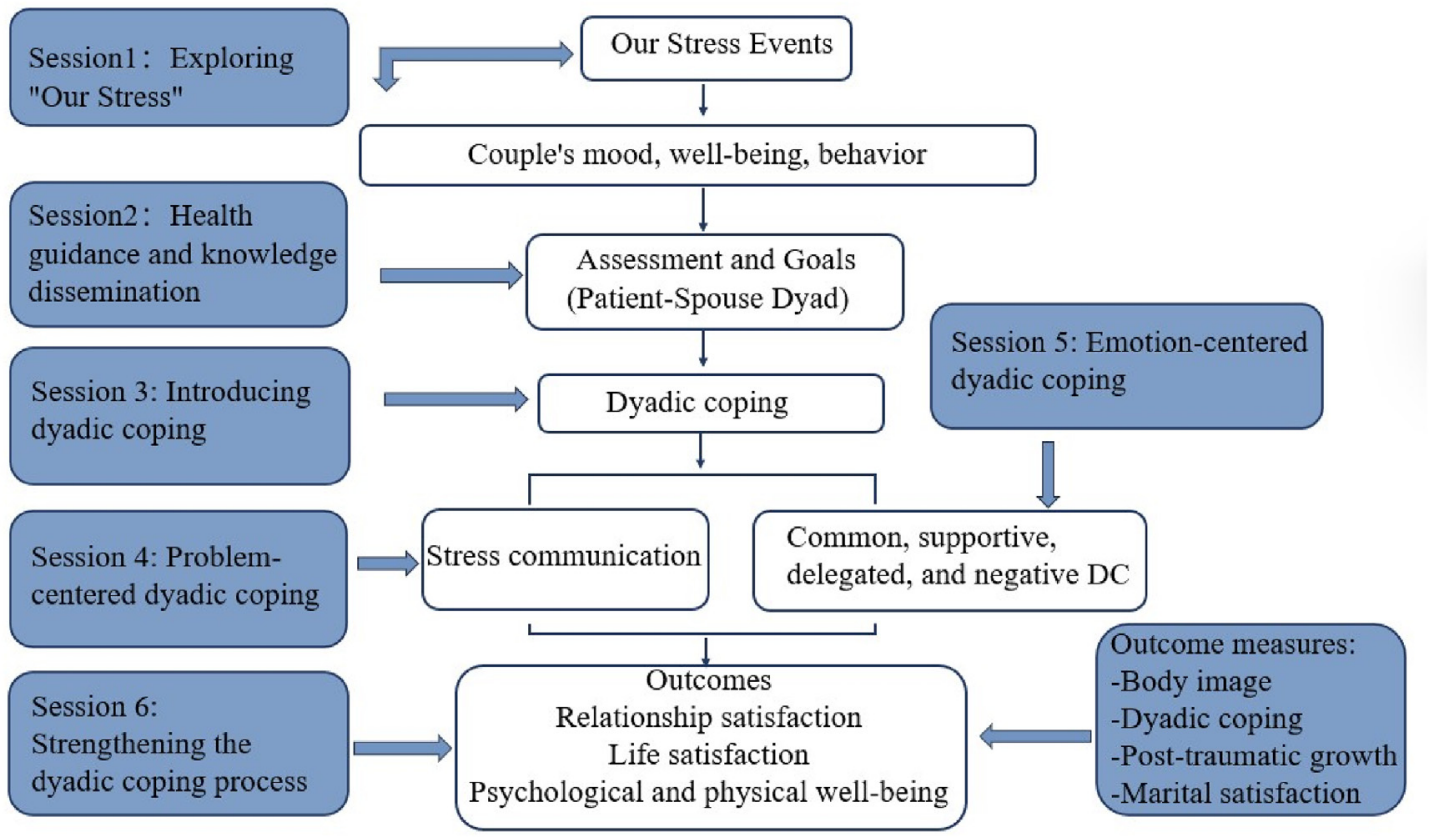
Systemic transactional model (STM) for dyadic coping in couple dyads (Adapted from Bodenmann, G. 1995).

The intervention program used in this study was developed based on a systematic trading model of dyadic coping (Fig. 1). Based on the theoretical framework of the model, we developed a six-session conference intervention program with interventions focused on both psychoeducation and skills training. The topics of the six-session intervention included: (1) exploring "our stress"; (2) health guidance and knowledge dissemination; (3) introducing dyadic coping; (4) triggering the dyadic coping process (problem-centered dyadic coping); (5) touching the dyadic coping process (emotion-centered dyadic coping); and (6) reinforcing the dyadic coping process. (See Supplementary Table 1 for session topics, outlines, and details.). In this study, BC couples were treated as a whole, and all intervention sessions were offered individually to BC couples. Due to the individual baseline differences of each couple, the researcher personalized the intervention content for each couple. Participants were free to withdraw from this study at any time.

The researchers used a combination of face-to-face and telephone sessions to implement the sessions. The duration of each session was approximately 60–90 minutes, depending on the situation.^30^ The sessions were intended to be conducted once a week for a set period of time. However, given the convenience of the participants' time, in practice the sessions were conducted at a frequency of between twice a week and once every two weeks.

### Measure

#### Demographic characteristics

The demographic and clinical information questionnaire was designed by the researchers and consisted of two parts: demographic information and clinical information. It included questions on age, education level, age at marriage, work status, religion, tumor stage, per capita monthly household income, current treatment received and duration of illness and cumulative hours of care.

#### Feasibility and acceptability

Recruitment rates (number of eligible couples enrolled in the program) and retention rates (number of couples completing the entire program) were calculated to assess the feasibility of the intervention program. The acceptability of the program was assessed using the Client Satisfaction Questionnaire (CSQ-8), which assesses satisfaction with the services received.^29^ Scores for each item on the CSQ-8 range from 1 to 4, with a maximum score of 32. A score below 21 indicates that participants were dissatisfied with the services they received. In this study, Cronbach's alpha coefficients were 0.86 for patients and 0.83 for spouses. Three open-ended questions were also asked in order to obtain more in-depth feedback from couples on this intervention program. These questions included "What parts of the program were most and/or least helpful?" and "What do you see as the limitations of this intervention?" and "What suggestions do you have for improving the intervention?". The CSQ-8 and open-ended questions were administered at the same time as the post-intervention evaluation.

#### Effectiveness of the intervention

Intervention effects were measured by effect sizes of whether four psychosocial health outcomes were meaningful as a result of the intervention. The primary outcomes were the level of body image and post-traumatic growth, and the secondary outcomes were level of dyadic coping and marital satisfaction.

(1) Body image self-rating questionnaire for breast cancer (BISQ-BC)

The body image self-rating questionnaire for breast cancer (BISQ-BC) revised by Jinghua An et al.^31^ was used to assess the patients' body image status. The BISQ-BC was compiled by Chinese scholar Kaina Zhou et al., in 2018.^32^ The revised version has 5 dimensions and 26 entries, including body image-related behavioral changes (7 entries), body image-related sexual activity (4 entries), body image-related role changes (5 entries), body image-related psychological changes (8 entries), and body image-related social changes (2 entries), and the entries are scored using a Likert 5-point scale, with a total score ranging from 26 to 130, with higher scores indicating a greater degree of body image disturbance in the patient. In this study, the Cronbach's alpha for this scale was calculated to be 0.797.

(2) Post-Traumatic Growth Inventory (PTGI)

The scale was developed by Tedeschi et al.^28^, ^33^ in 1996 using the Post-Traumatic Growth Inventory developed by scholars such as Wang et al.^34^ in 2011. The scale mainly evaluates the level of positive changes in post-traumatic breast cancer patients, which mainly consists of 5 dimensions of relating to others, new possibilities, personal strength, spiritual change, and appreciation of life, with a total of 20 entries. The scale is rated as 0–5 Likert 6 with a total score of 0–100, with higher scores representing better levels of post-traumatic growth. Cronbach's alpha coefficient in this study was 0.809.

(3) Dyadic Coping Inventory (C-DCI)

The Dyadic Coping Inventory (C-DCI) was developed by Bodenmann et al., in 2008, Chineseized and cross-culturally adapted by Xu et al.^35^ in 2016. Prof. Bodenman also divided dyadic coping into negative and positive dyadic coping, which is used to measure couples' shared reactions and coping styles in response to stressful events. The scale consists of 37 items divided into five dimensions: stress communication (8 entries), supportive coping (10 entries), empowering coping (4 entries), negative coping (8 entries), and common coping (5 entries). The last two entries serve as an overall evaluation of coping styles and are not included in the total score. Each item was scored on a 5-point Likert scale (1 ​= ​"rarely" to 5 ​= ​"often"). Higher scores indicate a more positive dyadic coping style. For this study, a Cronbach's alpha of 0.765 was calculated for this scale.

(4) Marital satisfaction (RDAS)

This study assessed participants' marital satisfaction using the Revised Dual Adjustment Scale (RDAS).^36^ The RDAS is a 14-item scale with six items designed to assess dyadic consensus, with scores ranging from 0 (completely disagree) to 5 (completely agree), four items assessing dyadic satisfaction, with scores ranging from 0 (all the time) to 5 (never), and the remaining four items assessing dyadic cohesion that ranging from 0 (never) to 4/5 (more often). Marital satisfaction scores on the RADS range from 0 to 69, with scores above 48 indicating high satisfaction. In this study, Cronbach's alpha for this scale was calculated to be 0.847 for patients and 0.835 for spouses.

## Data analysis

First, descriptive statistics were used to characterize sample characteristics, feasibility, acceptability, and outcome changes. Cronbach's alpha was used to calculate internal consistency of the scales. Given the limited data set, we employed the Shapiro–Wilk test to assess the normality of the samples. The results yielded consistent evidence in support of the assumption of normality. Then, paired t-tests were used to compare pre- and post-differences in psychosocial health outcome measures for patients and spouses, respectively; statistical significance was set at *P* ​≤ ​0.05. Finally, given the small sample size, we calculated effect sizes calculated by Cohen's d (large effect size, *d* ​= ​0.80; medium effect size, *d* ​= ​0.50; small effect size, *d* ​= ​0.20).^37^ In addition, the minimum clinically important difference (MCID) was calculated to assess changes in participant outcomes before and after the intervention. The small sample size also limited the efficacy of stratified analyses; therefore, we did not conduct multivariate analyses to examine the independent relationship between the outcome variable and the dependent variable. All analyses were performed using SPSS 26.0.

## Results

### Feasibility of recruitment and retention

**Fig. 2:**
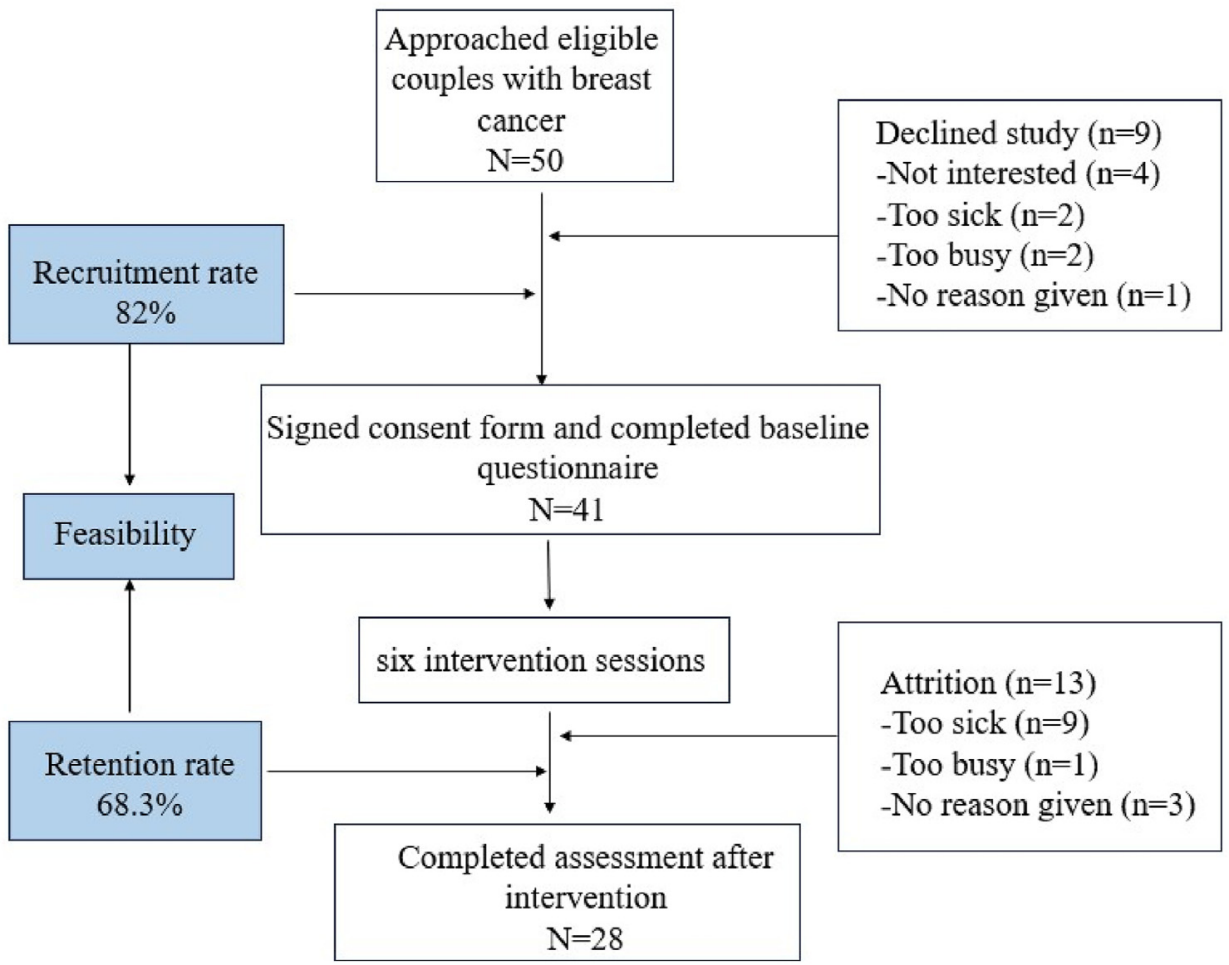
Consort diagram illustrating flow of participants into study.

The study flow of participants into the program is shown in Fig. 2. Of the 50 eligible BC couples approached by the researcher, 9 declined to participate in the study for the following reasons: one of the couple's partners was not interested in the program (*n* ​= ​4), the patient had a serious medical condition (*n* ​= ​2), too busy with no extra time (*n* ​= ​2), and no reason (*n* ​= ​1). As a result, 41 BC couples agreed to participate in the intervention and completed the baseline questionnaire, a recruitment rate of 82%. 28 couples received the six-session couples' dyadic coping-based intervention and completed the post-intervention assessment (retention rate of 68.3%). Couples withdrew because they were too busy to continue participating in the intervention (*n* ​= ​9), the patient's serious illness (*n* ​= ​1), or the intervention offered did not meet their expectations (*n* ​= ​3). In the 28 breast cancer patient-partner dyads, the patient's mean (SD) age was 50.64 years (range 40–62) and the spouse's mean (SD) age was 51.79 (range 43–64). 35.7% of the couples were married for more than 10–30 years; 14.3% of the patients were in high school or secondary school level education and 7.1% of the spouses were in high school or secondary school level education. 57.1% of the couples had per capita monthly household income at an average economic level (RMB 2000 to 5000 per month). 3 patients were in Stage I (10.7%), 14 in Stage II (50.0%), 10 in Stage III (35.7%), and 10 in Stage IV (25.6%), and 1 patient in stage IV (3.6%). Other participant characteristics are shown in Table 1.

**Table 1:** Demographic characteristics of patients with breast cancer.

Variable	Patients (*n* ​= ​28)	Spousal caregivers (*n* ​= ​28)
Age (years)	50.64 (40–62)	51.79 (43–64)
Education		
Primary school	7 (25.0)	4 (14.3)
Junior high school	14 (50.0)	19 (67.9)
High/secondary school	4 (14.3)	2 (7.1)
Junior college	1 (3.6)	3 (10.7)
Bachelor and above	2 (7.1)	0 (0.0)
Marriage length (years)		
< 10	2 (7.1)	2 (7.1)
10–30	10 (35.7)	10 (35.7)
> 30	16 (57.1)	16 (57.1)
Cumulative care hours (month)		
< 1 month		4 (14.3)
1-6 months		10 (35.7)
6-12 months		6 (21.4)
＞12 months		8 (28.6)
Occupation		
Incumbency	17 (60.7)	23 (82.1)
Unemployed	6 (21.4)	2 (7.1)
Retired	5 (17.9)	3 (10.7)
Religious beliefs		
No	26 (92.9)	26 (92.9)
Buddhism	2 (7.1)	2 (7.1)
Other	0	
Family monthly income per capita (Chinese yuan/RMP)		
＜2000	4 (14.3)	4 (14.3)
2001**–**5000	16 (57.1)	16 (57.1)
5001**–**10,000	7 (25.0)	7 (25.0)
＞10,000	1 (3.6)	1 (3.6)
Medical insurance		
Rural cooperative medical services	5 (17.9)	
Medical insurance for urban residents	14 (50.0)	
Out-of-town medical insurance	1 (3.6)	
Employee medical insurance	8 (28.6)	
TNM stage		
I	3 (10.7)	
II	14 (50.0)	
III	10 (35.7)	
IV	1 (3.6)	
Time of illness		
< 3 months	10 (35.7)	
3**–**6 months	8 (28.6)	
6**–**12 months	7 (25.0)	
1–3 years	1 (3.6)	
3**–**5 years	1 (3.6)	
＞5 years	1 (3.6)	
Current or previous treatment modality		
Surgery ​+ ​radiotherapy	25 (89.3)	
Surgery ​+ ​chemotherapy	1 (3.6)	
Surgery ​+ ​chemotherapy ​+ ​radiotherapy	2 (7.1)	
Transfer		
Yes	4 (14.3)	
No	24 (85.7)	
With or without children		
Yes	26 (92.9)	
No	2 (7.1)	

TNM, tumor-node-metastasis.

### Acceptability

Among BC couples who completed the couple-based dyadic coping intervention, the mean program satisfaction rating for patients was 24.36, standard deviation (SD) ​= ​3.93, and the mean program satisfaction rating for spousal caregivers was 23.68, SD ​= ​4.14. In response to an open-ended question, participants expressed a relatively high level of satisfaction with the program overall. Health guidance and knowledge dissemination were rated as the most useful aspects of the program. Participants reported that the knowledge provided in the health guide led to significant improvements in diet and exercise, and in addition, they suggested that they were able to maintain a positive attitude when coping with cancer-related problems. At the same time, couples emphasized that they increased communication and intimacy with each other by practicing emotion-focused dyadic coping and problem-focused dyadic coping strategies, reduced suspicion between the couples, and were particularly helpful in terms of the patient's body image and fostered a level of post-traumatic growth in both partners through dyadic behaviors. Program limitations reported by participants were primarily the difficulty participants with low literacy rates had in reading and understanding the guidelines. Some participants suggested that adding educational videos as supplemental materials to the intervention might help them better understand and reinforce the content of the intervention.

### Preliminary effect

Table 2 demonstrates pre- and post-intervention means and standard deviations for psychosocial health outcomes for breast cancer patients and spousal caregivers. For patients, the data suggests that small to moderate effects were significant in improving levels of body image (*d* ​= ​0.5, *P* ​= ​0.001) and post-traumatic growth (*d* ​= ​0.5, *P* ​= ​0.001). Small to moderate effects were also shown to be significant in dyadic coping (*d* ​= ​0.4, *P* ​= ​0.022) and marital satisfaction (*d* ​= ​0.5, *P* ​= ​0.001). The MCID results indicated that 50% of the patients clinically improved their level of body image after the intervention, and 39% clinically improved their level of post-traumatic growth after the intervention. 42%–68% of patients demonstrated clinically important differences in dyadic coping and marital satisfaction (Table 3). For spousal caregivers it could be observed that there was a small to moderate effect significant in improving post-traumatic growth (*d* ​= ​0.6, *P* ​< ​0.001); similarly, small to moderate effects were shown to be significant in dyadic coping (*d* ​= ​0.4, *P* ​= ​0.033) and marital satisfaction (*d* ​= ​0.3, *P* ​= ​0.021). Additionally, MCID results showed that 57% of spousal caregivers clinically improved post-traumatic growth after the intervention, and 36%–46% of spousal caregivers also demonstrated clinically important differences in dyadic coping and marital satisfaction (Table 3).

**Table 2:** Means, standard deviations, and effect sizes of study variables.

Outcomes	Patients (*n* ​= ​28)			*P*	Spousal caregivers (*n* ​= ​28)			*P*
	M (SD)				M (SD)			
	Pre	Post	Effect sizes Cohen's *d*		Pre	Post	Effect sizes Cohen's *d*	
Dyadic coping								
Dyadic coping inventory	107.0 (18.8)	115.5 (20.5)	0.4	0.022	110.1 (19.3)	118.4 (17.5)	0.4	0.033
Dyadic outcomes								
Body image	75.9 (13.7)	83.6 (17.0)	0.5	0.001	**-**	**-**	**-**	**-**
Post-traumatic growth	61.9 (13.7)	68.9 (11.3)	0.5	0.001	56.0 (10.2)	62.8 (11.6)	0.6	＜0.001
Marital satisfaction (RDAS)	35.1 (10.6)	40.4 (9.9)	0.5	0.001	36.9 (9.1)	40.2 (9.3)	0.3	0.021

M, Mean; SD, standard deviations; RDAS, Revised Dyadic Adjustment Scale.

**Table 3:** The MCID calculation and percentage of participants achieving MCID in outcomes.

Reported outcomes	ES		R (Measure reliability)	SD_baseline_		ES method^a^		SEM method^b^		Weighted MCID^c^		Achieving clinically important differences (pre-to-post intervention) [*n* (%)]	
	P	SC		P	SC	P	SC	P	SC	P	SC	P	SC
Dyadic coping inventory	0.4	0.4	0.78/0.75 (P/SC)	18.8	19.3	7.5	7.7	8.8	9.7	8.2	8.7	19 (68%)	13 (46%)
Body image	0.5	–	0.79	13.7	–	6.8	–	6.3	–	6.6	–	14 (50%)	–
Post-traumatic growth	0.5	0.6	0.79	13.7	10.2	6.8	6.1	6.3	4.8	6.6	5.5	11 (39%)	16 (57%)
Marital satisfaction (RDAS)	0.5	0.3	0.85/0.84 (P/SC)	10.6	9.1	5.3	2.7	4.1	6.7	6.0	4.7	12 (42%)	10 (36%)

DCI, Dyadic Coping Inventory; RDAS, Marital satisfaction; ES, Effect Size; MCID, Minimal Clinically Important Differences; P, Patients; SC, Spousal Caregivers; SD_baseline_, Standard Deviation of baseline; SEM, Standard Error of Measurement.

The distribution-based approach (ES method and SEM method) was used to calculate the MCID.

a ES method calculated MCID as ES ​× ​SD baseline.

b SEM method calculated MCID as 1 ​× ​SD baseline ​× ​(1-r) 1/2, r is the reliability of the measuring instrument.

c Weighted MCID ​= ​50% ∗ ES method a MCID+50% ∗ SEM method b MCID.

## Discussion

The present study validated the efficacy of STM-guided dyadic coping-based nursing interventions for patients with breast cancer and their spouses. Furthermore, it demonstrated that a diagnosis of breast cancer has profound physical and psychological consequences for both patients and spousal caregivers. The study also revealed that a dyadic coping intervention for couples had a more pronounced effect on enhancing the level of body image and post-traumatic growth of breast cancer patients compared to individual coping. This, in turn, enhanced spousal intimacy and provided new insights for future nursing interventions.

Our research has shown that an STM-guided dyadic coping-based nursing intervention is feasible and acceptable for patients with breast cancer and spousal caregivers. First of all, the intervention was feasible: a total of 41 couples participated in the study, of which 28 completed the entire intervention program, for a retention rate of 68.3%. Second, the intervention had good acceptability: follow-up surveys showed that participants reported good user satisfaction. Thirdly, the intervention showed initial efficacy: there were statistically significant post-intervention differences in levels of body image (patients), post-traumatic growth, dyadic coping, and marital satisfaction (couples). Effect sizes ranged from 0.3 to 0.6, i.e., small to moderate effects were significant.

Our study showed that the STM-guided dyadic coping intervention achieved better initial results in terms of patients' body image levels, reflecting the need for partners to play a supportive role in improving patients' body image. Previous studies have shown that patients with low levels of body image are reluctant to share their thoughts and feelings with their husbands due to the development of negative mentalities such as low self-esteem and stereotypes, which causes them to experience disconnection and suspicion and hinders dyadic behaviors between couples. Therefore, in our study, special emphasis was placed on the interactive behaviors that triggered couples' coping processes: problem-centered couples' coping and emotion-centered couples' coping, which play a more important role in enhancing couples' emotions. Second, couples indicated in semi-structured interviews that encouraging each other to speak their innermost thoughts can reduce suspicion. In terms of post-traumatic growth, 57% of spousal caregivers achieved better intervention outcomes before and after the intervention, compared to only 39% of patients. This may be due to the fact that patients with lower levels of body image have a harder time recovering from trauma,^38^ and that patients face side effects from treatments along with financial pressures and psychological burdens that demotivate them to recover from trauma.^39^ Our results also showed that couples also achieved a favorable intervention effect on the secondary outcome, marital satisfaction, which is consistent with previous research findings.^22^

Although the intervention yielded preliminary clinical outcomes, the attrition rates and semi-structured interviews of patients with breast cancer who participated in the program offer insights that can inform subsequent nursing interventions. Subsequent to the interviews, it became evident that a considerable number of couples had expressed reservations regarding the allocation of time between them. Consequently, the efficacy of the dyadic coping process (Sessions 4 and 5) may have been compromised. In light of this, the research team undertook an amendment to Sessions 4 and 5. Furthermore, couples indicated that they enhanced their communication and intimacy with each other through the utilization of emotion-focused and problem-focused dyadic coping strategies. These approaches were observed to diminish suspicion between the couples, prove beneficial for the patients' level of body image, and facilitate the level of post-traumatic growth in both partners through dyadic behaviors. The primary issue reported by participants in relation to the program was the difficulty encountered by those with low literacy rates in reading and comprehending the guidelines. Some participants proposed that the incorporation of educational videos as supplementary resources to the intervention could have facilitated a more comprehensive understanding and reinforcement of the intervention's content. The primary challenge encountered in this pilot intervention was the recruitment of patients and their spousal carers. It was observed that there were instances where one partner expressed interest while the other did not. Given that couples often interact with each other, future researchers may wish to consider focusing on the member of the couple who is more interested in participating in the intervention.^40^ This approach may encourage the other partner to participate, thus improving recruitment rates for dyadic interventions.

In this preliminary study, patients appeared to benefit more than spouses, particularly in terms of dyadic coping and marital satisfaction, which is consistent with the results of the Colorectal Cancer Couples Intervention.^41^ Under the stress of illness, patients need support and therefore may be more in need of access to the knowledge or skills provided by the intervention.^26^ However, for spousal caregivers, due to their caregiver role, they are more focused on providing support to the patient at the expense of their own health.^19^

In summary, this intervention not only provides supportive information and coping strategies for the patient, but also for the spousal caregiver, allowing both to increase their understanding of the illness and their confidence in personal coping. In particular, the interventions provide BC couples with psychological regulation and emotional management, improve the patient's body image, and enable couples to recognize the nature of the “unity” and to become more involved in couple behavior in an effort to overcome difficulties.^42^ Furthermore, enhancements in DC can result in enhanced cancer adjustment for patients and their spousal caregivers, as evidenced by improvements in body image, marital satisfaction, and post-traumatic growth. It is anticipated that future developments will see the creation of additional STM-guided couple-based DC interventions, which will prove beneficial for couples affected by other types of cancer.^43^ However, the initial study design lacked a control group, thereby preventing any conclusion that the observed effect was solely attributable to the intervention and allowing other factors (e.g., routine medical care) to be considered as potential contributors.^44^ Furthermore, the limited sample size precludes the possibility of testing the mediating effect of DC on cancer adaptation outcomes. A larger sample size would be required to test the effect of the intervention relative to the control condition, for example, through the use of a randomized controlled trial. Furthermore, a larger sample would be necessary to validate the mediating effect of DC on the outcome of cancer adaptation in couples.

### Limitations

This pilot study has several limitations. Firstly, there was no control group, so we cannot be sure whether the changes observed in this trial were the result of the intervention or of other factors. This pilot study focused on assessing the feasibility, acceptability, and initial efficacy of the intervention; therefore, future studies will need to test the efficacy of their interventions through rigorous randomized controlled trials. Second, all findings are based on self-reports and may be biased. Thirdly, the follow-up survey was conducted within one week of the intervention, which did not allow for observation of long-term effects, and future studies should consider extending the follow-up period to verify long-term efficacy. Fourthly, our small sample size did not allow us to conduct stratified analyses or examine independent relationships between intervention effects and key covariates (e.g., demographics), and thus the sample size should be expanded in the future. Fifthly, validation was only conducted in a Chinese population, which may limit the generalizability of the results to populations from other cultures, and more studies should be conducted in the future to validate the effectiveness of this intervention in different cultural contexts.

## Conclusions

Despite recognized research limitations, the meaningful results of this pilot study have some potential implications for future practice. In breast cancer care, the patient and spouse should be viewed as a disease community, with close attention paid to the physical and psychological effects on the patient, as well as the spousal caregiver. Encouragement to promote conjugal behavior in a positive way for both partners; in addition, there is a need to enhance the assessment of the patient's body image, so that health care professionals can develop targeted interventions, which in turn will promote intimacy and marital satisfaction for both partners.

## Ethics statement

The study design was approved by the Ethics Committee of the Affiliated Hospital of Jiangnan University in Wuxi, Jiangsu Province, China (IRB No. LS2023073). All participants provided written informed consent.

## CRediT authorship contribution statement

W.F.S and W.M.H contributed to the conception of the study, Y.W contributed to the conception of the study, performed the data analyses and wrote the manuscript, J.J.Z, S.W, Y.B.W and L.H collected the data. All authors had full access to all the data in the study, and the corresponding author had final responsibility for the decision to submit for publication. The corresponding author attests that all listed authors meet authorship criteria and that no others meeting the criteria have been omitted.

## Funding

This work was supported by the Supports for 10.13039/100018696Leading Talents in Medical and Health Profession (Mading academician, 4532001THMD), Beijing Bethune charitable Foundation (Grant No. 2022-YJ-085-J-Z-ZZ-011), Top Talent Support Program for Young and Middle-aged people of Wuxi Health Committee (Grant No. BJ2020047). The funders had no role in considering the study design or in the collection, analysis, interpretation of data, writing of the report, or decision to submit the article for publication.

## Data availability statement

Data sets generated and/or analyzed during the current study are not publicly available but may be obtained from the corresponding authors upon reasonable request.

## Declaration of generative AI and AI-assisted technologies in the writing process

No AI tools/services were used during the preparation of this work.

## Declaration of competing interest

The authors declare no conflict of interest.
