# Supplementary material for: Feasibility and preliminary efficacy of a systematic transaction model-guided dyadic coping nursing intervention for patients with breast cancer and their spousal caregivers: A pilot study
Source: Asia Pac J Oncol Nurs. 2024 Nov 14;12:100621. doi: 10.1016/j.apjon.2024.100621 (PMC11664281; doi:10.1016/j.apjon.2024.100621)
Supplement: Multimedia component 1 [file mmc1.docx]

Supplementary Table 1. Main contents, outline, and dyadic outcome measures of the program

| **session** | **contents** |
| --- | --- |
| **1.Exploring "Our Stress"** | -Introduces the research team and provides an overview of the program.  - Couple profiles: learn about the couple's demographics and background information.  -Breast Cancer Body Image Self-Assessment Questionnaire.  -Post-traumatic growth.  -Dyadic Coping Scale.  -Marital satisfaction.  -Identify the challenges posed by breast cancer.  -Impact the couple's mood, well-being, and behavior.  -Discusses facing cancer-related stress and discusses the couple's concerns, mental state, and coping styles.  *Homework: Discuss with your spouse how you feel about the way you are coping with cancer; think about where you need more supportive information.* |
| **2.Health guidance and knowledge dissemination** | -Provide materials and education (guidelines) on breast cancer:  (1) Distribute breast cancer knowledge brochures (including breast cancer causes, diagnosis, chemotherapy adverse effects and management, complications, functional exercises, etc.).  (2) Share relevant functional exercise videos and rehabilitation instruction videos. Allow patients and spouses to jointly evaluate the goals of postoperative functional exercise and learn functional exercise drills following the videos.  (3) Provide dietary guidance.  -Propose strategies for maintaining a positive attitude when coping with cancer-related problems:  (1) Assess psychological changes before and after the disease (both spouses).  (2) Encourage the adoption of a positive mindset, e.g., positive stress relief, outdoor exercise, developing a hobby.  *Homework: Read the educational pamphlet; discuss the benefits of this session for you and your partner.* |
| **3.Introducing dyadic coping** | -Review of the content of the last meeting.  -Emphasis on "Our Disease": the impact of cancer on couples, both physiologically and psychologically.  -Presentation of material on dyadic coping: definition, content and meaning of binary coping (guidelines).  -Discusses everyday dyadic coping behaviors adopted by couples in coping with cancer.  -Share thoughts and feelings about "our disease" and dyadic coping.  *Homework: read educational pamphlets; discuss areas of coping together;* |
| **4. Trigger the dyadic coping process (problem-centered dyadic coping)** | -Reviewed from last meeting.  -Focus on the body to confront the disability (cognitive appraisal, problem coping).  -Discuss the frequency, style, and content of communication between partners in daily life.  -Distribute to the patient and spouse an outline manual of nursing interventions, including daily interactions and emotional communication between the couple.  (1) Cognitive evaluation: ① Assess the patient's problems with body image through the results of the Body Image Chinese Version of Breast Cancer Questionnaire (BIBCQ-C) questionnaire, which the patient completed for the first time.  ② Encourage patients and their spouses to state the changes in the patient's body and their respective feelings. ③ Ask the patient and his/her spouse about the difficulties they encountered in coping with the patient's physical changes.  (2) Problem response: Targeted guidance for the problems in the patient's questionnaire: hair loss is temporary, and wigs or hats can be worn; unhealed flap sites will grow back after mastectomy; prosthetic breasts can be worn; make-up guidance can be given to those whose eyebrows and eyelashes have fallen off, and to those with obvious hyperpigmentation; nutritional guidance can be given to those with large changes in their body shape, and so on. Help patients and their spouses to solve the difficulties encountered in the process of coping in a timely manner. Instructing patients in arm rehabilitation exercises and telling them and their spouses to adhere to effective functional exercises can promote the functional recovery of the affected limbs and accelerate the recovery of body shape. |
| **5. tactile dyadic coping process (emotion-centered dyadic coping)** | -Review the content of the last meeting.  -Practice effective communication skills:  (1) Self-disclosure and communication of the patient and spouse, guiding one party to make active disclosure and the other party to listen carefully and respond appropriately. For example, guide both parties to tell the good memories that have happened together, such as the scene of the first acquaintance, the achievements obtained by the children, the difficulties successfully overcome, etc. The time of disclosure is decided according to the patient's wishes.  (2) Spouse's attentive companionship. Explain to the patient's spouse the importance of accompanying the patient with heart and specific practices. Accompanying with the patient with heart is mainly divided into: a. Courage to take the responsibility. Encourage spouses to take the initiative to learn breast cancer related knowledge, observation and treatment of adverse reactions to chemotherapy; take care of the patient's daily life, such as accompanying for checkups, delivering meals, laundry, etc.; and actively undertake the social responsibility of comforting other family members and coordinating work, etc. b. Accompany the patient. Instruct spouses to accompany patients. When the patient's physical condition permits, accompany the patient for walks, rehabilitation exercises and other activities; prepare small gifts for the patient on a regular basis; in addition, when the patient is in a bad mood, the spouse should patiently comfort and encourage the patient. c. Spiritual communication. Instruct the spouse to regulate his/her own emotions, maintain a good mental state, and communicate with each other in a celebratory tone more often, such as celebrating the timely discovery, celebrating the surgery, etc. d. Accept the change of each other's body image. Praise and encourage the other person, name more of the other person's strengths, appreciate the other person's past efforts, and express praise sincerely.  -Introduce materials (guides) on the negative effects of dyadic coping.  -Reflect on personal experiences and feelings related to negative binary coping.  -Share effective coping skills; |
| **6. Strengthening the dyadic coping process** | -Overview the entire program to the patient and spouse and reinforce the program.  -Assessing binary outcomes: body image, post-traumatic growth, dyadic coping, and marital satisfaction.  -Conducting semi-structured interviews to collect open-ended questions.  -Completing a client satisfaction questionnaire; |
